# MAR Elements and Transposons for Improved Transgene Integration and Expression

**DOI:** 10.1371/journal.pone.0062784

**Published:** 2013-04-30

**Authors:** Déborah Ley, Niamh Harraghy, Valérie Le Fourn, Solenne Bire, Pierre-Alain Girod, Alexandre Regamey, Florence Rouleux-Bonnin, Yves Bigot, Nicolas Mermod

**Affiliations:** 1 Institute of Biotechnology, University of Lausanne, and Center for Biotechnology UNIL-EPFL, Lausanne, Switzerland; 2 Selexis SA, Geneva, Switzerland; 3 PRC, UMR INRA-CNRS 7247, Centre INRA Val de Loire, Nouzilly, France; 4 GICC, UMR CNRS 7292, UFR de Médecine, Tours, France; Montana State University, United States of America

## Abstract

Reliable and long-term expression of transgenes remain significant challenges for gene therapy and biotechnology applications, especially when antibiotic selection procedures are not applicable. In this context, transposons represent attractive gene transfer vectors because of their ability to promote efficient genomic integration in a variety of mammalian cell types. However, expression from genome-integrating vectors may be inhibited by variable gene transcription and/or silencing events. In this study, we assessed whether inclusion of two epigenetic control elements, the human Matrix Attachment Region (MAR) 1–68 and X-29, in a piggyBac transposon vector, may lead to more reliable and efficient expression in CHO cells. We found that addition of the MAR 1–68 at the center of the transposon did not interfere with transposition frequency, and transgene expressing cells could be readily detected from the total cell population without antibiotic selection. Inclusion of the MAR led to higher transgene expression per integrated copy, and reliable expression could be obtained from as few as 2–4 genomic copies of the MAR-containing transposon vector. The MAR X-29-containing transposons was found to mediate elevated expression of therapeutic proteins in polyclonal or monoclonal CHO cell populations using a transposable vector devoid of selection gene. Overall, we conclude that MAR and transposable vectors can be used to improve transgene expression from few genomic transposition events, which may be useful when expression from a low number of integrated transgene copies must be obtained and/or when antibiotic selection cannot be applied.

## Introduction

Efficient gene transfer and expression for functional studies, protein production or gene and cell therapies usually requires reliable DNA delivery and transcription into target cells. Gene transfer methods based on viral and non-viral vectors have been developed to maximize gene delivery and expression, but an expression system combining high levels of reliability, efficacy and safety is currently lacking. For instance, non-viral vectors are associated with a reduced risk of insertional mutagenesis when compared to e.g. retroviral vectors for gene or cell therapies, and they are easier to produce [1]. However, they typically require physical (e.g. electroporation) or chemical (e.g. cationic lipids) DNA transfer methods that are not easily applied in vivo, and they are less efficient than viral vectors when genomic integration of the transgene is necessary.

Genome integration is usually a requisite for persistent transgene expression in dividing cells. Integration can be mediated by cellular activities when plasmid vectors are used. For instance, stable transfection relies on the selection of rare cells having integrated plasmid DNA into one or few genomic loci, as a result of the action of cellular DNA repair and recombination enzymes [2]. This leads to the integration of multi-copy plasmid concatemers, usually as head-to-tail arrays [3], [4]. However, repetitive transgene arrays are prone to unstable expression, especially when gene amplification methods are applied, which can result in variable transgene expression or silencing [5]. Thus, epigenetic regulatory elements are often added to plasmid vectors to alleviate such unfavorable effects, and very high levels of expression can therefore be obtained from cultured cells lines *in vitro* [6]. Nevertheless, the integration of many transgene copies can complicate the screening of cell lines producing recombinant proteins for pharmaceutical use. Indeed, it is expected to increase the probability of observing point mutations in one or few copies, which are often difficult to detect during early cell line characterization stages, and it has been associated to repeat-induced silencing events [5]–[7].

Alternatively, DNA recombination enzymes such as transposases, viral integrases, or synthetic integrases may be expressed transiently in target cells or introduced together with the transgene-bearing DNA to assist transgene integration. This usually yields increased frequencies of transgene integrations when compared to plasmid vectors. Among these are the proteins mediating targeted genomic DNA cleavage, such as the meganucleases and zinc finger nucleases that allow DNA integration in particular genomic loci, in contrast to the more random integration events mediated by viral integrases and transposases [8]. However, targeted integration usually occurs in a subset of the cells only, and it results in the integration of one or two transgene copies at the most, which limits expression. Recombinases and nucleases can also mediate non-specific DNA cleavage events and chromosomal rearrangements [9], which limits their use to in vitro cultured cells.

Among non-viral vectors, transposons are particularly attractive because of their ability to integrate single copies of DNA sequences with high frequency at multiple loci within the host genome [10]. Unlike viral vectors, some transposons were reported not to integrate preferentially close to cellular genes, and they are thus less likely to introduce deleterious mutations. Moreover, transposons are readily produced and handled, consisting of a transposon donor plasmid containing the cargo DNA flanked by inverted repeat sequences and of a transposase-expressing helper plasmid or mRNA. Several transposon systems were developed to mobilize DNA in a variety of cell lines without interfering with endogenous transposon copies. For instance, the piggyBac (PB) transposon originally isolated from the cabbage looper moth [11] efficiently transposes cargo DNA into a variety of mammalian cells [12].

Epigenetic regulatory elements can be used to protect the transgene from unwanted epigenetic effects when placed near the transgene on plasmid vectors. For example, elements called matrix attachment region (MARs) were proposed to increase transgene genomic integration and transcription while preventing heterochromatin silencing, as exemplified by the potent human MAR 1–68 [2], [13], [14]. They can also act as insulators and thereby prevent the activation of neighboring cellular genes [15]. MAR elements have thus been used to mediate high and sustained expression in the context of plasmid or viral vectors [16]. However, whether these favorable properties of the MAR elements may be combined to those of transposable vectors remains essentially untested, and their potential effects on transposition efficacy and/or transgene expression when placed within a transposon remain unknown. Here, we evaluated the use of a piggyBac transposon containing human MARs in CHO cells. We show that MARs may be included in transposon vectors to mediate efficient and sustained expression from a few transgene copies, using cell populations generated without an antibiotic selection procedure.

## Results

### Effect of MAR Inclusion on Transposition Efficiency

Transposition efficiency varies significantly depending on the transposon system and cell type. Therefore, we first determined optimal transfection conditions for our model CHO cells. Different quantities and ratios of plasmid bearing the puromycin resistance-carrying transposon and of the PB transposase expression vector were co-transfected into cultured cells, and the occurrence of antibiotic-resistant colonies were scored (Figure S1A and S1B), as performed in earlier studies [17], [18]. The frequency of puromycin-resistant colonies was increased up to 5-fold in the presence of the transposase, when compared to spontaneous genomic integration of the plasmids upon co-transfections with a PB-devoid control plasmid (Figure S1C and data not shown). This indicated that the PB transposase was very likely functional in CHO cells.

**Figure 1:**
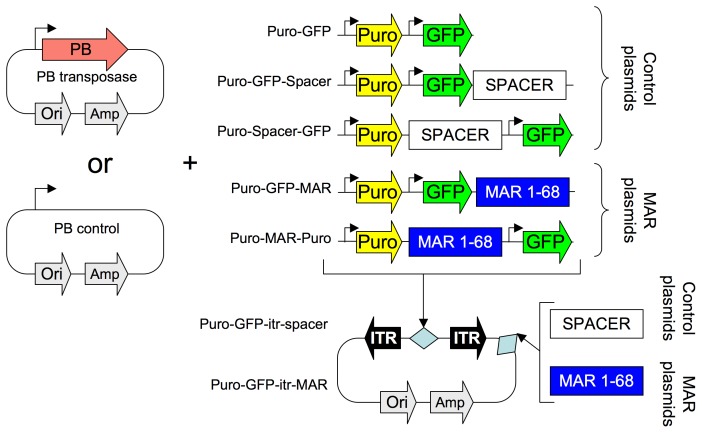
Overview of the GFP expression constructs used in this study. The piggyBac inverted terminal repeats (ITR), the transposase coding sequence (PB), the bacterial origin of replication (Ori) and the ampicillin selection gene (Amp) schematically depict the plasmids used in this study. The puromycin resistance (Puro) and reporter green fluorescent protein (GFP) genes are illustrated by yellow and green arrows and the spacer or MAR 1–68 sequences are shown by white or blue boxes on the transposon donor plasmid derivatives.

As antibiotic resistance does not necessarily reflect efficient transgene expression, we next used the green fluorescent protein (GFP) expressed from a strong GAPDH cellular promoter derivative as an indicator. To test whether adding a MAR element to the PB transposon may affect transposition efficiency and transgene expression, and to assess whether the location of the MAR in the construct had any influence on these effects, we designed a series of transposon donor constructs containing the GFP and puromycin resistance (Puro) gene, in which the MAR 1–68 or a control neutral spacer DNA sequence were inserted at different positions in the plasmid (Figure 1). The parental Puro-GFP transposon plasmid without an insert was used as a control of transposition, to distinguish the impact of increased transposon size relative to effect of the MAR or spacer sequence addition.

**Figure 2:**
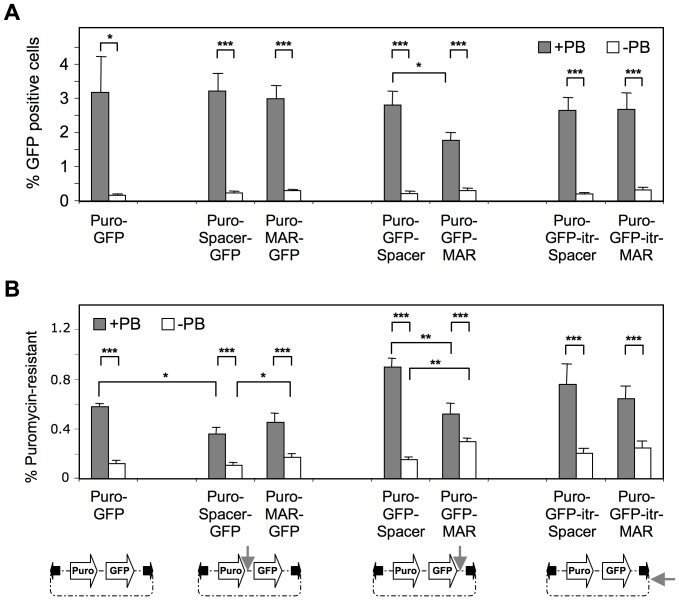
Effect of MAR and transposon size on transposition efficiency. CHO cells were co-transfected with 300 ng of Puro-GFP, Puro-MAR-GFP, Puro-GFP-MAR or Puro-GFP-ITR-MAR plasmids, or with their respective control plasmids containing the spacer, with 300 ng PB transposase (filled boxes) or control plasmids (open boxes). Grey arrows on the plasmid schematic representations indicate the position of the MAR or spacer DNA, while black arrows depict PB’s ITR. (**A**) Cells were cultured for 3 weeks after transfection without selection pressure and the percentage of GFP-expressing cells was quantified by cytofluorometry. (**B**) Puromycin-resistant colonies from 50 000 transfected cells were counted after 2 weeks with puromycin selection. The percentage of cells leading to puromycin resistant colonies was determined. Values represent the means ± SEM (n = 3). *P<0.05, **P<0.01, ***P<0.001.

We first estimated the transposition efficiency of the various transposon constructs by assessing the percentage of GFP-expressing cells after transfection and three weeks of cultivation without antibiotic selection, so as to dilute away and eliminate non-integrated and thus non-replicating episomal plasmids. In parallel, we also assessed transposition efficiency by counting puromycin-resistant colonies as before. When using the parental Puro-GFP transposon vector, cytofluorometry analysis indicated that approximately 3% of the cells stably expressed GFP when the transposase was expressed, whereas less than 0.2% retained detectable expression from spontaneous genomic integration resulting from cellular recombination activities (Figure 2A and Figure S2). The MAR or spacer sequence did not alter the occurrence of cells stably expressing GFP from transposition events when included downstream of the puromycin resistance coding sequence but upstream of the promoter and enhancer driving GFP expression (Figure 2A, Puro-MAR-GFP vs Puro-spacer-GFP constructs, filled columns). However, the MAR significantly inhibited transposition when placed at the transposon extremity next to one of the inverted terminal repeats (ITR), but not when it was placed on the external side of the ITR, just outside of the transposed sequences (Figure 2A, Puro-GFP-MAR vs. Puro-GFP-itr-MAR), suggesting topological constraints to the transposition mechanism.

Antibiotic selection yielded a lower apparent transposition frequency from the parental Puro-GFP construct (3% vs. 0.6%, Figure 2A and 2B), implying that transposition does not always allow expression of the puromycin resistance gene at levels that are sufficiently high to mediate cell survival, while detection of low GFP expression levels can be detected by cytofluorometry. Apart from this difference, similar conclusions were reached, i.e. a centrally located MAR did not alter significantly the occurrence of transposition events, while MAR inclusion at the edge of the transposon inhibited transposition when compared to the control containing a neutral spacer sequence. Inclusion of the MAR either upstream or downstream of the GFP coding sequence also increased the occurrence of antibiotic resistant colonies by spontaneous plasmid integration in the absence of the transposase, as observed in previous studies (Figure 2B, open columns) [13]. However, this effect was more pronounced when scoring antibiotic resistance than GFP fluorescence, indicating that it may be attributed in part to the occurrence and selection of a minority of highly-expressing and antibiotic-resistant cells obtained in presence of the MAR element.

### Effect of MAR Inclusion on Expression from Transposed Genes

**Figure 3:**
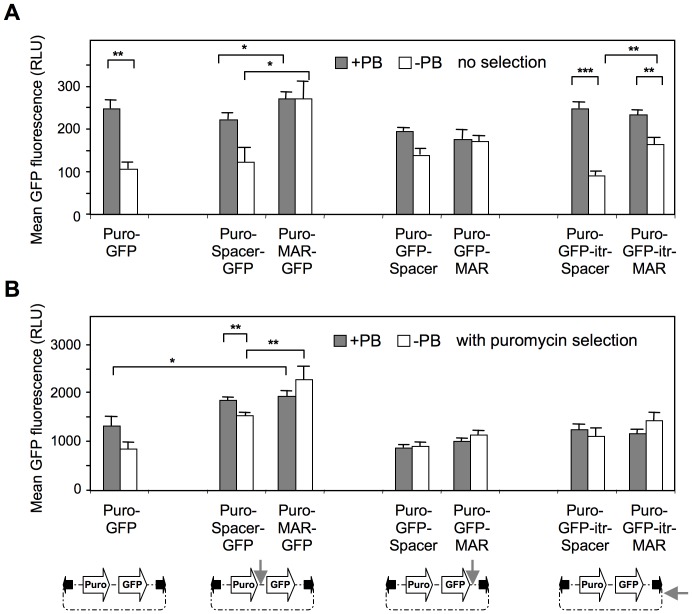
Effect of MAR on transgene expression levels. The average GFP fluorescence of the cells as described in the legend of Figure 2 was quantified by cytofluorometric analysis, either after 3 weeks of culture without selection following transfection (**A**), or after 2 weeks under puromycin selection (**B**). Values represent the means ± SEM (n = 3). *P<0.05, **P<0.01, ***P<0.001. RLU : relative light unit.

Apart from transposition efficiency, another aspect of transposable vectors is the level of expression they allow. This was analyzed by probing the GFP fluorescence levels of the CHO cells cultured for 3 weeks with or without selection for puromycin resistance, and with or without transposase expression, taking into account the fluorescence of GFP-positive cells only, so that variable transposition or genomic integration frequencies did not influence expression values. In the absence of the transposase and without puromycin selection, the MAR significantly increased expression when placed upstream of the GFP transgene (Figure 3A), as expected from its known effect to improve transcription efficiency and to decrease silencing [19]. However, inclusion of MAR 1–68 just downstream of the GFP gene had little effect on expression, while placing the MAR further downstream restored some activation of GFP expression. This indicated that the relative positions and/or distance of the MAR and transgene expression elements can modulate the activation effect.

In the presence of the transposase, the highest level of GFP expression from unselected cells was observed when the MAR was centrally located, but not when the MAR was placed downstream of the GFP coding sequence, nor when inserted outside of the transposed sequence as expected (Figure 3A). In the presence of puromycin selection, the MAR-mediated activation was reduced, either with or without the transposase, while the GFP expression averages were increased by one order of magnitude (Figure 3B). This confirmed that puromycin selection yielded only the minority of the cells that display the highest expression levels, as proposed above from the quantitation of transposition events. It further indicated that the transposable vectors containing a centrally located MAR yielded similar expression levels when compared to their plasmid counterpart transfected without the transposase.

### Effect of MAR Inclusion on the Copy Number of Integrated Transposon

Higher GFP fluorescence levels may result from an increased transcription of the transgenes and/or by the integration of more transgene copies [2], [19]. This was assessed by quantifying the number of genome-integrated transgene copies resulting from the various types of vectors. Total genomic DNA was isolated from pooled populations of cells, either after cytofluorometric sorting of fluorescent cells from unselected populations or after selection for puromycin resistance. The transgene copy number was determined by quantitative polymerase chain reaction (qPCR) analysis of the GFP coding sequence relative to the cellular β2-microglobulin (B2M) gene. In the absence of antibiotic selection, the average number of transgenes integrated by either the transposase or by cellular recombination enzymes were similar, around 1–6 copies per genome, and they were not significantly affected by the MAR or control sequence (Figure S3A). However, the lowest copy number was obtained when the MAR was included at the transposon edge, supporting our earlier conclusion that it decreases transposition at this location. After selection for highly expressing cells with puromycin, the number of transposed transgenes was in a similar 2–7 copy range (Figure S3B). However, the number of transgenes copies integrated in the absence of the transposase was generally significantly higher, ranging from 6 to 14 copies. This can be readily explained by the fact that spontaneous integration usually results in the integration of concatemers of multiple plasmid copies at a single genomic locus (Figure S2), and that higher transgene copy numbers should lead to higher expression levels when cells subjected to silencing effects have been removed by antibiotic selection. Taken together with the prior conclusion that antibiotic selection preferentially yields highly expressing cells, this also indicated that spontaneous plasmid integration results in a more variable number of transgene copies than transposable vectors.

**Figure 4:**
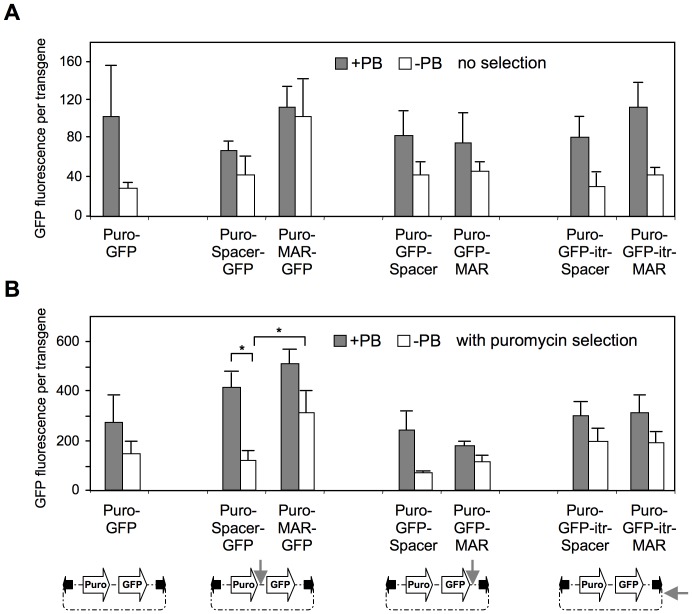
Effect of MAR on transposon copy number. The copy number of integrated GFP was determined using qPCR as described in supplementary. Fig S4, using unselected puromycin-resistant cells generated as described in the legends of Figure 2. The mean GFP fluorescence was divided by the number of integrated GFP transgene copies, to estimate the average expression per integrated GFP copy in unselected (**A**) and puromycin-resistant (**B**) CHO cells. Values represent the means ± SEM (n = 3). *P<0.05.

We then normalized GFP expression to the gene copy number to assess the intrinsic expression potential of the vectors, independently from their propensity to integrate in the genome. Overall, lower expression per transgene copy was obtained from unselected cells, or from antibiotic-selected cells transfected without transposase or centrally-located MAR, indicating that transgene expression is influenced both by the inclusion of the epigenetic regulatory element and by the mode of transgene integration (Figure 4). Expression per gene copy was generally increased by the transposase, when assessed from various vectors and combination of elements, and this was observed with or without antibiotic selection. The highest levels of expression per transgene copy were obtained after antibiotic selection from the cells generated with the transposon vector containing the MAR element centrally located and in presence of the transposase. Inclusion of the MAR immediately downstream of the GFP coding sequence did not increase transgene expression significantly, as noted earlier for the absolute levels of expression.

Finally, we assessed whether the favorable effect of MAR 1–68 on expression may be specific to the strong human GAPDH promoter used here, or whether it would also occur with other promoters. Thus we replaced the human GAPDH promoter driving GFP expression by the weaker simian virus 40 (SV40) early promoter. Use of the weaker promoter yielded comparable numbers of GFP-positive cells and of integrated transgenes, indicating that the transposition efficiency is not altered by transgene expression (Figure S4A and S4B vs. Figure 2A and S3B). However, the absolute levels of expression were lower with the SV40 promoter (Figure S4C vs. 3B). In addition, expression normalized to the transposon copy number was decreased by 4.6-fold by the use of the SV40 promoter in the absence of the MAR, and by 3.1-fold with MAR 1–68 (Figure S4D vs. 4B). This indicated that the MAR could partially, but not fully prevent the decrease of expression resulting from the use of a weaker promoter, even in presence of the transposase. Overall, we concluded that a few integrated copies are sufficient to obtain high transgene expression from transposons, and that the highest expression per transgene is obtained when MAR 1–68 is placed upstream of the strong promoter.

### Expression of Therapeutic Proteins from Transposon Vectors Devoid of Selectable Gene

We next wished to determine whether MAR-containing transposons may be used to express therapeutic proteins using the CHO-M cell line grown in suspension culture [2], and whether antibiotic selection may be dispensable. As cells growing in suspension are not efficiently transfected by lipofection agents, we first assessed CHO-M electroporation with the piggyBac vectors containing MAR 1–68. Electroporation increased the frequency of GFP expressing cells up to approximately 15% of the total cell population when using the vector with the centrally located MAR 1–68, and expression was stably maintained for over 4 weeks of culture without selection (Figure S5A). However, the presence of this MAR at the edge of the transposon decreased transposition efficiency, as observed earlier using cationic lipid-based gene transfer. The antibiotic selection gene was removed from this vector, and MAR 1–68 was replaced by another potent human element, MAR X-29 [13]. Inclusion of the MAR X-29 at this position restored a high proportion of GFP positive cells (Figure S5A).

**Figure 5:**
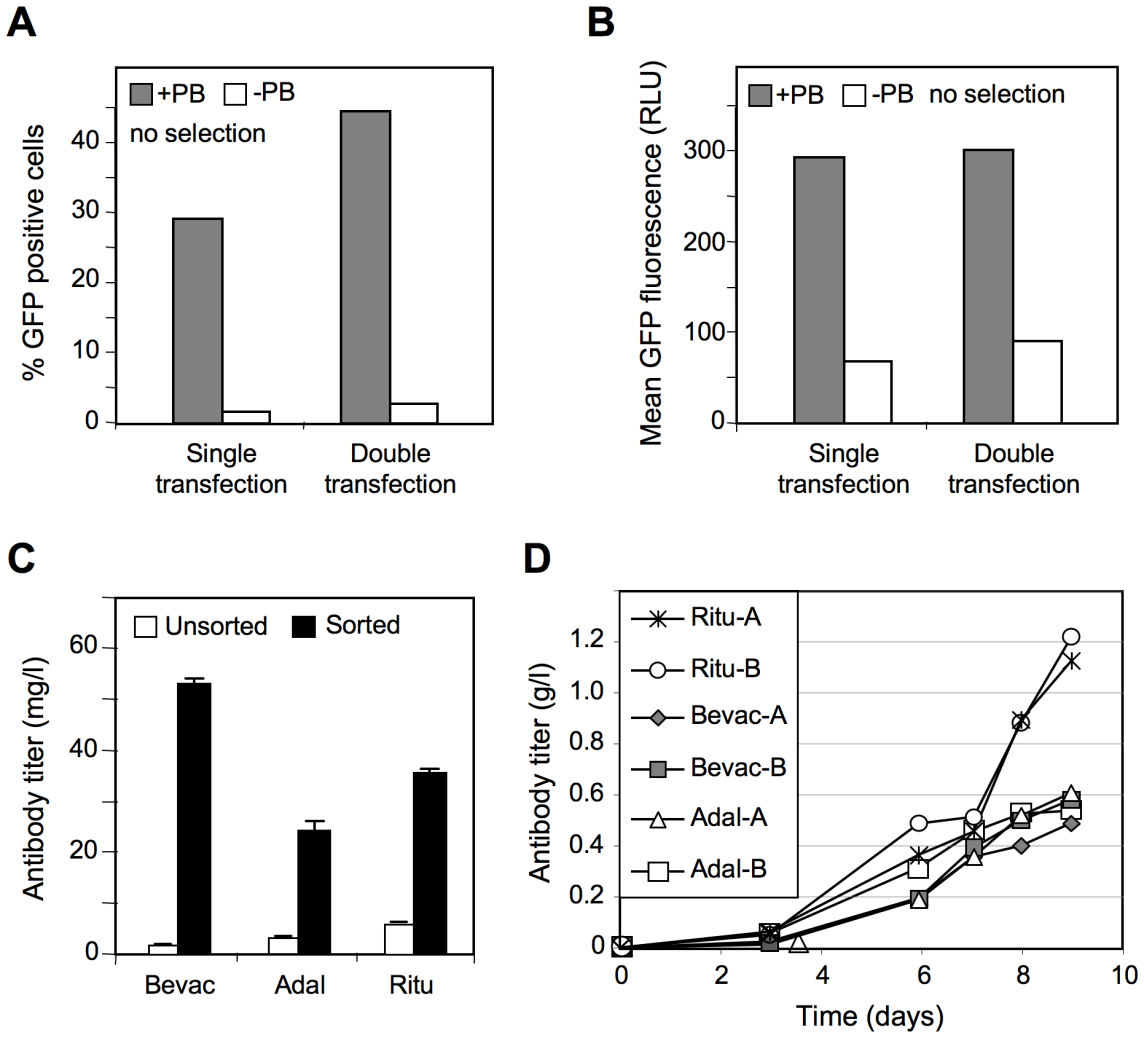
Recombinant protein expression from electroporated CHO-M cell suspensions. (**A**) CHO-M cell were electroporated once or twice with the MAR X-29-bearing GFP-expression transposon vector in the presence (+PB) or not (-PB) of the piggyBac transposase, as described in Figure S5, and the percentage of stable GFP-expressing cells was assayed after 3 weeks of culture performed in the absence of selection. (**B**) Mean of the GFP fluorescence of the GFP-positive cells. (**C**) cDNAs encoding immunoglobulin light and heavy chains of the Bevacizumab (Beva), Adalimumab (Adal) and Rituximab (Ritu) antibody were introduced in MAR X29-containing transposon plasmids instead of GFP. The light and heavy chain transposon constructs were electroporated three times at 12 days intervals with the piggyBac transposase expression vector in CHO-M cells, and the levels of immunoglobulin secreted in the culture supernatants of polyclonal cell pools grown without selection was assayed (open bars). Alternatively, the unselected polyclonal cell populations were sorted by panning cells displaying immunoglobulins at their surface using magnetic micro-beads, and the levels of secreted immunoglobulins were assayed as for the unsorted populations (closed bars). (**D**) Alternatively, immunoglobulin-expressing colonies were sorted from transfected cell populations using a colony-picking device, and two clones expressing each of the three immunoglobulins were grown in fed-batch cultures in spin-tube bioreactors. The levels of secreted immunoglobulins were determined as for panel (C).

CHO-M cells were electroporated once or twice with the single transgene MAR X-29-containing transposable vector, using a multiple gene transfer procedure [2], and the total cell populations were grown without selection for three weeks before cytofluorometry analysis. A higher proportion of fluorescent cells was recorded from two consecutive transfections performed in the presence of the transposase as compared to transfections performed without the transposase vector (Figure S5B). Electroporation yielded 30% and 45% of the cells stably expressing GFP upon one or two successive transposon vector electroporations respectively, which compares favorably to the 2–4% of positive cells obtained from the lipofection of adherent cells (Figure 5A vs. 2A). Overall, this indicated that the transposition efficiency was much higher after electroporation than with chemical transfection, possibly because a higher proportion of cells take-up and transiently express the transferred DNA when performing multiple electroporation procedures. However, transgene expression levels were similar, as the mean fluorescence levels of GFP-positive electroporated cells were comparable to those obtained after chemical transfection of the transposable vector (Figure 5B vs. 3A).

Expression cassettes encoding the heavy or light chains of three therapeutic immunoglobulins (Bevacizumab, Adalimumab and Rituximab) were inserted upstream of the MAR X-29 into the transposable vector instead of the puromycin resistance and GFP coding sequences. The resulting constructs were electroporated thrice with the piggyBac transposase vector into CHO-M cells. After three weeks of culture without selection, secretion of the three antibodies was quantified from the cell culture supernatants. Titers ranging from 1 to 8 µg/ml of Bevacizumab, Adalimumab and Rituximab were obtained in several transfection experiments, indicating that significant expression of these antibodies may be obtained when assessing the culture supernatants of unselected polyclonal cell populations (Figure 5C). These levels were further increased to 23–55 µg/ml by sorting the expressing cells using streptavidin-coated magnetic microbeads, so as to capture the cells that transiently display the secreted immunoglobulins at their surface [20]. This indicated that polyclonal cell populations may be sorted to display and express therapeutic proteins in amounts allowing analytical or functional studies, at levels similar to those previously obtained from transposable vectors after antibiotic selection [21].

We next assessed whether highly expressing cell clones may be obtained from the non-sorted and non-selected polyclonal populations using an imaging device for cell colony productivity. Two colonies displaying favorable size, indicative of fast cell growth, and high immunoglubulin secretion were picked from each of the three immunoglobulin-expressing cell populations. Immunoglobulin secretion by the 6 cell clones was assessed using small scale cell suspension cultures in spin tube bioreactors. The immunoglobulin-expressing clones produced antibody titers ranging between 0.55 to 1.2 g/L over the 9-day fed-batch cultures (Figure 5D), while reaching high cell densities and maintaining elevated viability (Figure S6). This indicated that transposable vectors can yield cell clones displaying the high titers, cell viability and cell density needed to produce proteins for therapeutic use.

**Figure 6:**
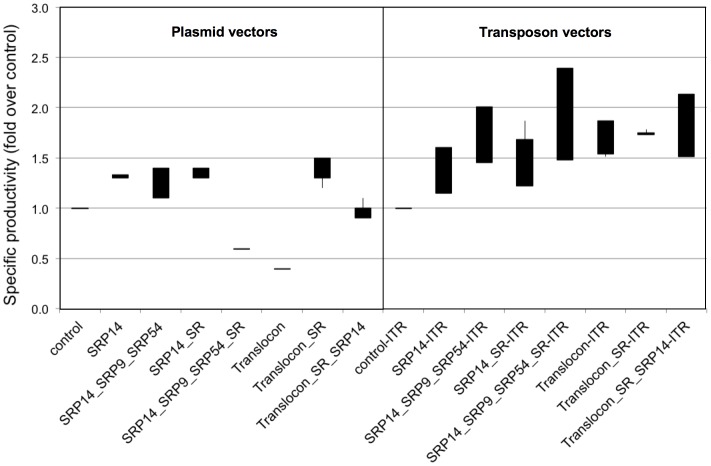
Effect of the expression of secretion proteins from transposable and plasmid vectors on recombinant protein expression. (**A**). Transposable or regular plasmid vectors were constructed to express secretion proteins SRP9, SRP14, SRP54, the SRP receptor a and b subunits (SR), or the Translocon. Transposable vectors were co-transfected with the piggyBac transposase vector, whereas the non-transposable plasmid vectors were transfected alone in a cell clone expressing the infliximab antibody [22]. After three weeks of culture without selection, the levels of secreted infliximab antibody were assayed from cell culture supernatants. Data pertaining to the effect of transposable vectors are as in [22].

We next assessed whether transposon vectors may also be used for the metabolic engineering of CHO cells to achieve improved secretion of therapeutic proteins. We have recently shown that the expression of difficult-to-express immunoglobulins like the Infliximab antibody can be increased significantly by the expression of proteins of the secretion pathway, such as the signal peptide recognition proteins SRP9, SRP14, SRP54, the SRPRalpha and SRPRbeta components of the SRP complex receptor (SR), and/or the Translocon subunits [22]. CHO-M cells stably expressing the Infliximab antibody were thus co-transfected either with a regular plasmid expression vector and with a neomycin selection plasmid, or with the transposase expression plasmid and transposable vectors expressing similar combinations of secretion proteins. Cells transfected with plasmid vectors were selected for spontaneous transgene integration into their genome by neomycin selection, whereas cells transfected with the transposable vectors were grown without selection in parallel. Cultures of these polyclonal populations were then assessed for specific antibody secretion per day and per cell. Immunoglobulin expression was found to be slightly improved, up to 1.5-fold relative to the control cells expressing Infliximab only, upon the expression of secretion proteins from plasmid vectors (Figure 6). However, higher secretion of the therapeutic protein was obtained from transposable vectors, with yields that were increased by more than two-fold upon the expression of several combinations of components of the protein secretion pathway. Thus, we concluded overall that transposable vectors can be used to increase the expression of recombinant proteins, either directly by bearing the therapeutic protein coding sequences, or indirectly, by allowing the expression of cellular proteins that improve the secretion of the therapeutic protein. Furthermore, the use of transposable vectors allowed easy integration of numerous transgenes into the cell’s genome, obviating the need for multiple antibiotic selection genes.

## Discussion

So far, most studies involving transposable vectors have relied on antibiotic resistance-based assays, which has not allowed a clear and quantitative disentanglement of transgene copy number-linked effects from those elicited by variations of transgene expression levels. In this study, we wished to evaluate the potential benefit of adding an epigenetic regulator to a transposable vector in conditions allowing the quantitative assessment of both transposition efficiency and transgene expression. This study shows that the human MAR 1–68 can be added at a central position in the piggyBac transposon without decreasing transposition efficiency, while it’s insertion at the transposon edge may interfere with the transposase function, both in terms of transposition efficiency, and, surprisingly, expression per transgene. Inclusion of the MAR 1–68 at the edge of the transposon may cause topological constraints that would hinder the joining of the two ITR by the transposase, which would in turn reduce transgene integration by the transposase. The decrease of expression per transgene copy elicited by MAR 1–68 when inserted at the transposon edge suggests that the transposons may have integrated at genomic loci that are less favorable for expression. Thus, the MAR 1–68 may have altered the mechanism by which the transposase targets a genomic locus to integrate the transposon sequence. Interestingly, this effect appears to be specific to MAR 1–68, as inclusion of human MAR X-29 at the edge of the transposable sequence did not yield low transposition efficiency or expression. While the cause of such MAR-dependent effects remains unclear, this may result from the specific organization of DNA bending-prone sequences as well as transcription factor binding sites whose combinations and relative positions differ when comparing distinct MAR elements [13].

Interestingly, the extent of the MAR-mediated activation of transposed genes was reduced when compared to that of spontaneous plasmid integration. Furthermore, the level of expression, when normalized to transgene copies, was higher from the transposons than those obtained from the spontaneous integration of the plasmids in the absence of the transposase. This effect was observed irrespective of the size of the constructs, of the presence of the MAR or of promoter strength. This would be expected if transposition might often occur at genomic loci that are relatively permissive for expression, for instance because open chromatin structures may be more accessible to both the transposase and transcription factors. In this respect, previous studies have suggested that transposons may preferentially integrate within gene introns [23], at promoters [24], or at genomic loci with lower propensity for silencing, although this has remained a matter of debate (see [25] for a review). Alternatively, the co-integration of many plasmid copies at the same genomic locus, as elicited by spontaneous integration events, may lead to the formation of heterochromatin and to the silencing of repetitive sequences, which the MAR would oppose, whereas single-copy transposon integration may be less prone to such chromatin-mediated silencing. In addition, the integration of transposons at multiple independent genomic loci makes it likely that at least one copy landed into a favorable genomic environment and is expressed, whereas plasmid integration was found to occur predominantly at just one genomic locus [13].

Further work may be required to determine why expression from plasmid vectors is more dependent on the MAR than transposon vectors. Nevertheless, our results indicate that the highest expression levels per transgene were obtained from a MAR-containing transposon when coupled to a strong promoter, and that high expression levels can be obtained from a few transposed transgene copies. Use of transposable vectors may be advantageous for the production of proteins for pharmacological use. For instance, fewer integrated transgene copies should be advantageous if high productivities can nevertheless be obtained, as documented in this study for three therapeutic antibodies, because it should decrease the probability of point mutation occurrence in one or in a subset of the transgenes, as elicited from spontaneous mutagenic events. In addition, transposase-mediated integration events may be less mutagenic than the DNA repair and recombination mechanisms involved in spontaneous plasmid integration, which can lead to incomplete or rearranged transgene copies.

The high efficiency of genomic integration by the piggyBac transposon should also be favorable when the amount of target cells is limiting, for instance for the non-viral transfer of therapeutic genes in primary stem cells to generate clonal populations, with cell-based therapies or regenerative medicine as perspectives. In this context, physiological expression levels from a few transposed gene copies and the frequent occurrence of transposition events, thus obviating the need for antibiotic selection, should be of advantage, since the use of antibiotic resistance genes and/or unreliable transgene expression may raise safety concerns.

Finally, transposons should also be favorable for cellular metabolic engineering, for instance to express secretion proteins and/or when multiple rounds of gene introduction are required. This is illustrated in this study by the expression of multiple proteins of the cell’s secretory pathway, where the transfection of multiple vectors and/or multiple successive transfection cycles may exhaust available antibiotic or other selection methods. Alternatively, the ability to quickly express therapeutic proteins without a need for antibiotic selection is of interest, for instance when multiple therapeutic protein candidates must be expressed for screening purposes, as significant amounts of proteins can be obtained from unselected cell populations 2–3 weeks after transfection. Overall, we thus conclude that MAR-containing transposons will be useful additions to the currently available arsenal of expression vectors.

## Materials and Methods

### Plasmids and DNA Vectors

The PB transposase expression vector pCS2+U5V5PBU3 contains the PB transposase coding sequence surrounded by the 5′ and 3′ untranslated terminal regions (UTR) of the Xenopus laevis β-globin gene. This plasmid was constructed as follows: the 3′ UTR 317 bp fragment from pBSSK/SB10 (kindly provided by Dr S. Ivics) was inserted into pCS2+U5 (Invitrogen/Life Technologies, Paisley, UK) to yield pCS2+U5U3. The PB transposase coding sequence (2067 bp, GenBank accession number: EF587698) was synthesized by ATG:biosynthetic (Merzhausen, Germany) and cloned in the pCS2+U5U3 backbone between the two UTRs. The PB control vector corresponds to the unmodified pCS2+U5 plasmid (Figure 1, left panel).

The different transposons vectors used in this study were generated by introducing the PB 235 bp 3′ and 310 bp 5′ inverted terminal repeats (ITRs), synthesized by ATG:biosynthetic (Merzhausen, Germany), into the pBluescript SK- plasmid (pBSK ITR3′-ITR5′, Figure 1, right panel). The puromycin resistance gene (Puro^R^), under the control of the SV40 promoter from pRc/RSV plasmid (Invitrogen/Life Technologies), was then inserted between the two ITRs. The MAR 1–68 and MAR X-29 elements, the puromycin resistance and GFP genes used in this study were as previously described [2], [13], [14]. The immunoglobulin expression vectors and the SRP9, SRP14, SRP54, SRPRalpha, SRPRbeta, SEC61A1, SEC61B and SEC61G coding sequences were as described by Le Fourn et al. [22]. A DNA spacer of 3.6 kb corresponding to the MAR 1–68 length was PCR-amplified from the mouse utrophin cDNA and used as control without MAR. The GFP, immunoglobulin or secretion proteins were expressed using a eukaryotic expression cassette composed of a human CMV enhancer and human GAPDH promoter upstream of the coding sequence followed by a SV40 polyadenylation signal, the human gastrin terminator and a SV40 enhancer [22]. For transposition experiments from a weak promoter (Figure S6), the human GAPDH promoter was replaced by the SV40 promoter. Expression cassettes and/or MAR elements were inserted between the ITR sequences or in the bacterial vector backbone as illustrated in Figure 1 and in figure legends using standard cloning methods. All plasmids, DNA vectors, and other renewable resources, as represented in Figure 1, will be made freely available for non-profit research use, unless specifically restricted by some other party.

### Cell Culture and Transfection Analysis

The CHO DG44 cell line [26] was cultivated in DMEM: F12 (Gibco) supplemented with Hypoxanthine/Thymidine (HT, Gibco) and 10% fetal bovine serum (FBS, Gibco). Transfections were performed using PEI (JetPRIME, Polyplus Transfection), according to the manufacturer’s instructions. Cells were transfected with various amounts of a supercoiled plasmid encoding the PB transposase (ranging from 0 to 1500 ng) for titration experiments or co-transfected with the optimal ratio of 300 ng of PB transposase expression plasmid and 300 ng of transposon donor plasmid. Supercoiled transposon-donor plasmids were used, as it was shown that PB transposition from linear vector is relatively inefficient when compared to circular transposon plasmids [18]. Plasmids transfected as controls without the transposase were also supercoiled, as the use of linearized plasmids did not increase significantly the frequency of occurrence of spontaneous integration (<2-fold, data not shown). Two days after the transfection, cells were transferred to several Petri dishes depending on the experiment. For analysis of unselected transfected CHO cells, cells were replated without antibiotic selection for 3 weeks and the percentage of fluorescent cells and the fluorescence intensity of GFP positive cells were determined by FACS analysis using a CyAn ADP flow cytometer (Beckman Coulter). For gene copy number analysis of unselected cells, stable GFP positive CHO cells were sorted using a FACSAriaII. For antibiotic resistant colony-counting assays, 50,000 transfected cells were seeded in 100 mm plates and selected with 5 µg/ml puromycin for 2 weeks. Then, resistant colonies were fixed and stained in 70% EtOH 0,7% Methylene Blue for 10 min, and colonies >0.5 mm in diameter were counted. For GFP expression studies, cells were selected for two weeks before GFP fluorescence FACS analysis as described above.

CHO-M cells were maintained in suspension culture in SFM4CHO Hyclone serum-free medium (ThermoScientific) supplemented with L-glutamine (PAA, Austria) and HT supplement (Gibco, Invitrogen life sciences) at 37°C, 5% CO2 in humidified air. Transposon donor plasmids were transferred in these cells by electroporation according to the manufacturer’s recommendations (Neon devices, Invitrogen). Quantification of immunoglobulin secretion was performed from batch cultures as described previously [22]. Briefly, cell populations expressing immunoglogulins were evaluated in batch cultivation into 50 ml minibioreactor tubes (TPP, Switzerland) at 37°C in 5% CO2 humidified incubator for 7 days. Immunoglobulin concentrations in cell culture supernatants were measured by sandwich ELISA.

### qPCR Gene Copy Number Assays

Total DNA was isolated from CHO stable cell pools following transposition assays using the DNeasy Tissue Kit (Qiagen, Hilden, Germany) according to the manufacturer’s protocol. The copy number of genome-integrated transgenes was assessed using 6 ng of genomic DNA by quantitative PCR using the SYBR Green-Taq polymerase kit from Eurogentec Inc and ABI Prism 7700 PCR machine (Applied Biosystems). The GFP-Forward: ACATTATGCCGGACAAAGCC and GFP-Reverse: TTGTTTGGTAATGATCAGCAAGTTG primers were used to quantify the GFP gene, while primers B2M-Forward: ACCACTCTGAAGGAGCCCA and B2M-Reverse: GGAAGCTCTATCTGTGTCAA were used to amplify the Beta-2 microglobulin gene. For the amplicon generated by the B2M primers, one hit was found per CHO haploid genome after alignment to our CHO genome assembly using NCBI BLAST software. As CHO are near-diploid cells [27], we estimated that B2M is present at 2 copies per genome. The ratios of the GFP target gene copy number were calculated relative to that of the B2M reference gene, as described previously [28].

### Sorting and Assay of Immunoglobulin-expressing Cells

To magnetically sort IgG-expressing cells, transfected CHO-M cells were seeded at a cell density of 3×10^5^ cells per ml in SFM4CHO medium (Thermo Scientific) supplemented with 8 mM L-glutamine and 1× HT supplement (both from Gibco), referred to as Complete Medium. After 4 days in culture, 2×10^6^ cells were washed, re-suspended in PBS and incubated with a biotinylated human IgG (KPL216-1006) at a final concentration of 3 µg/ml, together with 30 µl pre-washed MyOne T1 streptavidin-coated Dynabeads (Invitrogen), on a rotary wheel for 30 minutes at room temperature. The cell and bead mix was then placed on a magnet to separate labeled cells from non-labeled cells. The beads were washed 4 times with a phosphate buffer saline (PBS) solution. After the final PBS wash, the beads and cells were re-suspended in 500 µl pre-warmed Complete Medium, transferred to a 24 well plate and incubated at 37°C with 5% CO_2._ After 24 h the magnetically-sorted polyclonal cells were separated from the beads and incubation was continued until the cells were of a sufficient density for expansion in 50 mL TPP spin tube bioreactors (Techno Plastic Products AG, Switzerland).

Alternatively, two clones were isolated from non-sorted and non-selected populations expressing each of the three IgGs using a ClonePix device. Briefly, semi-solid media was used to immobilize single cells, and colonies secreting high amounts of IgG were picked ten days post-embedding. These cell lines were passaged every 3–4 days in spin tube bioreactors at a density of 3×10^5^ cells/ml in a peptone-containing growth medium (Hyclone SFM4CHO supplemented with 8 mM glutamine) in a humidified incubator maintained at 37°C and 5% CO2, with orbital shaking at 180 rpm.

IgG titers were determined from cells seeded at a cell density of 1×10^5^ cells per ml and grown for 6 days in 5 ml of Complete Medium in 50 ml Spin tube bioreactors when assessing polyclonal cell populations. Alternatively, shake flask cultures of clonal populations were inoculated at a density of 3×10^5^ cells/ml into SFM4CHO media to initiate the fed batch production process. Fed batch production assays were performed with 25 ml of culture volume in 125 ml shake flasks or 5 ml in 50 ml TPP culture tubes in humidified incubators maintained at 37°C and 5% CO2 with shaking at 150 rpm (125 ml shake flask and spin tubes). The production was carried out for ten days by feeding 16%, of the initial culture volume of chemically defined concentrated feed (Hyclone, Cell Boost 5, 52 g/l) on days zero, three and six to eight. No glutamine and glucose feeding was applied during the culture run. The viability and viable cell density (VCD) of the culture was measured daily using a GUAVA machine (Millipore). A double sandwich ELISA assay was used to determine MAb concentrations secreted into the culture media.

## Supporting Information

Figure S1 **Optimization of piggyBac transfection conditions in CHO cells.** PB transposition activity was measured under fixed amount of the puromycin resistance gene bearing transposon plasmid (300 ng) co-transfected with increased amount of transposase plasmid. (A) Puromycin resistant colonies were stained after 2 weeks of selection. (B) Fold increase in colony number induced by the PB transposase. Values represent the means ± SD (n = 2).
(TIFF)

Figure S2 **Models of transgene integration from transfected plasmids.** (A) Model of multi-copy plasmid chromosomal integration resulting from classical stable transfection methods, resulting in a preferential head-to-tail transgene organization [3], [4]. (B) Mode of excision of a transposon (flanked by ITR sequences) from a donor plasmid, and single-copy integration at one or several loci in the host genome by the transposase.
(TIFF)

Figure S3 **Effect of MAR and transposase upon integrated transgene copy number.** The number of integrated GFP transgene copies was determined using qPCR, and values were normalized relative to the cellular B2M gene, using genomic DNA isolated from unselected CHO cells (A), or puromycin-resistant cells (B) generated as described in the legends to Figs 2 and 3. Values represent the means ± SEM (n = 3). *P<0,05.
(TIF)

Figure S4 **Effect of MAR 1–68 on transposition efficacy and transgene expression from a weak promoter.** CHO cells were transfected as described in the legend to Figure 2 using transposon donor constructs containing a centrally located spacer or MAR sequence, as indicated, except that the GAPDH promoter driving GFP expression was replaced by the SV40 promoter. The percentage of GFP positive cells (A) was determined from unselected cells as for Figure 2A, whereas the GFP transgene copy number normalized to the cellular betamicroglobulin gene (B), the mean cellular fluorescence (C), and the GFP fluorescence normalized to the GFP transgene copy number (D), were determined from puromycin-selected cells, as described for Figure S3, 3B and 4B, respectively. Values represent the means ± SEM (n = 3). *P<0,05, **P<0.01, ***P<0.001.
(TIF)

Figure S5 **Assay of single transgene MAR-bearing transposable vectors.** (A) CHO-M cells were electroporated with the depicted transposon donor constructs containing or not human MAR 1–68 or X-29 together with the piggyBac transposase expression vector. The percentage of GFP positive cells was determined from the total cell population maintained in suspension culture for 12 or 33 mean population doubling time. (B) Densitometric profiles of CHO-M electroporated with the GFP-MARX-29 transposon donor with (+PB) or without (-PB) the piggyBac transposase expression vector. Cell fluorescence was assayed after single or double transfections followed by 3 weeks of cell culture performed in the absence of selection. The horizontal bar indicates the sectors used to quantify expressing from non-expressing cells.
(TIFF)

Figure S6 **Culture growth of cell clones producing therapeutic antibodies from transposable vectors.** The viable cell densities (A) and the cell viability (B) are shown for the tube spin bioreactor runs displayed in Figure 5D for the indicated cell clones (labeled A or B) expressing the Bevacizumab (Beva), Adalimumab (Adal) or Rituximab (Ritu) antibody.
(TIF)
