# Supplementary figures and images for: MAR Elements and Transposons for Improved Transgene Integration and Expression
Source: PLoS One. 2013 Apr 30;8(4):e62784. doi: 10.1371/journal.pone.0062784 (PMC3640020; doi:10.1371/journal.pone.0062784)

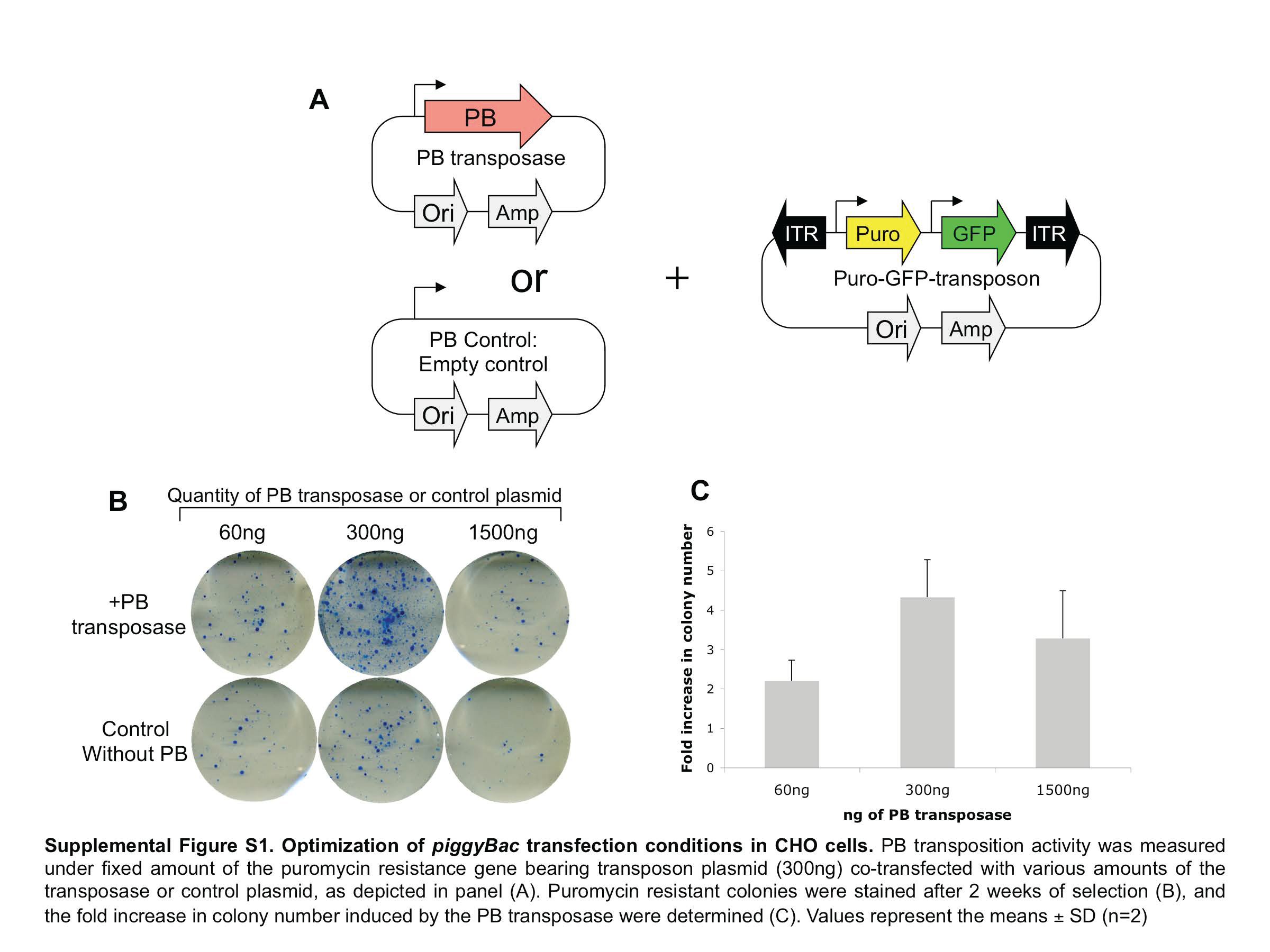

Supplement: Figure S1 — Optimization of piggyBac transfection conditions in CHO cells. PB transposition activity was measured under fixed amount of the puromycin resistance gene bearing transposon plasmid (300 ng) co-transfected with increased amount of transposase plasmid. (A) Puromycin resistant colonies were stained after 2 weeks of selection. (B) Fold increase in colony number induced by the PB transposase. Values represent the means ± SD (n = 2). (TIFF) [file pone.0062784.s001.tiff]

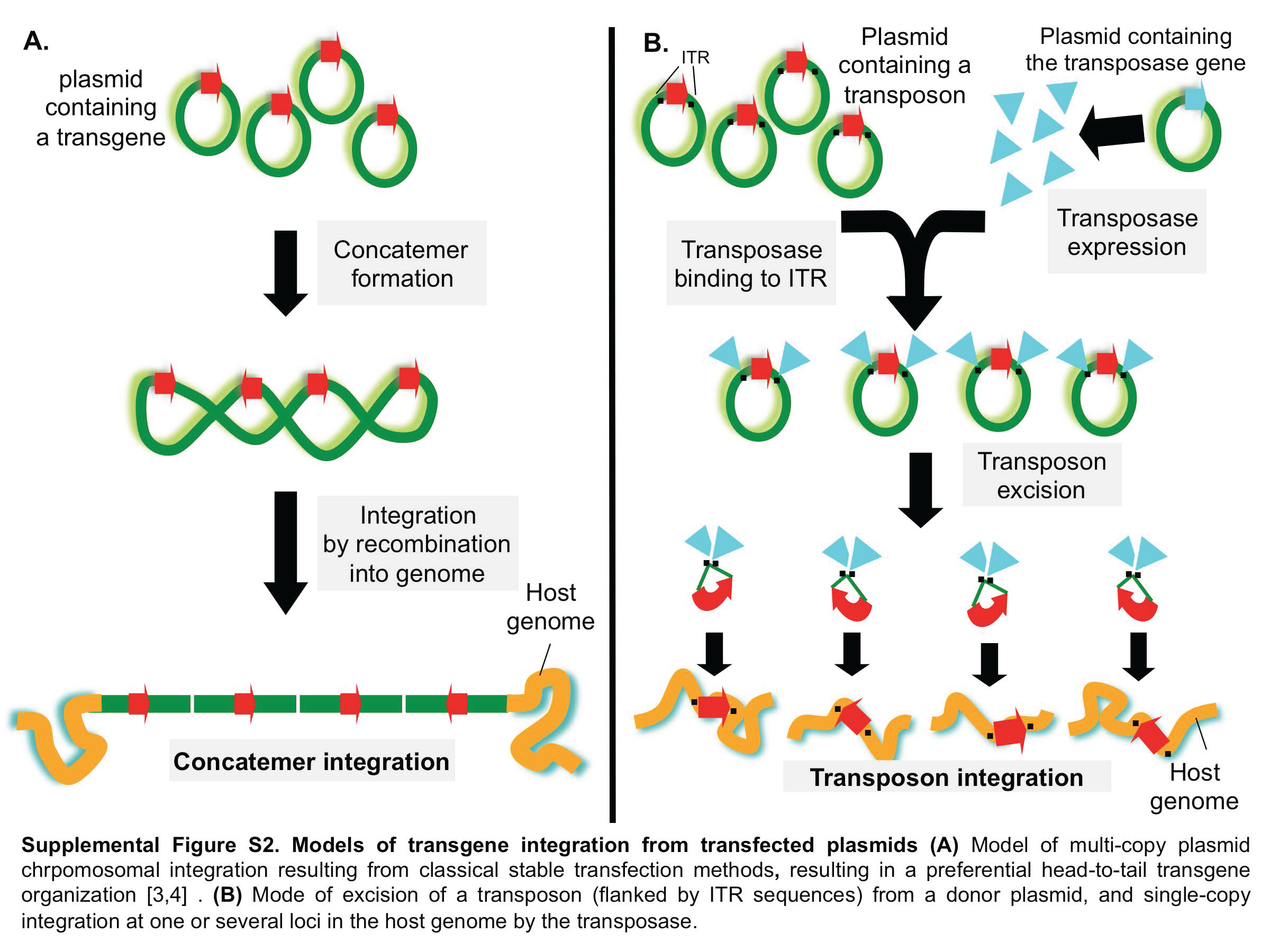

Supplement: Figure S2 — Models of transgene integration from transfected plasmids. (A) Model of multi-copy plasmid chromosomal integration resulting from classical stable transfection methods, resulting in a preferential head-to-tail transgene organization [3], [4]. (B) Mode of excision of a transposon (flanked by ITR sequences) from a donor plasmid, and single-copy integration at one or several loci in the host genome by the transposase. (TIFF) [file pone.0062784.s002.tiff]

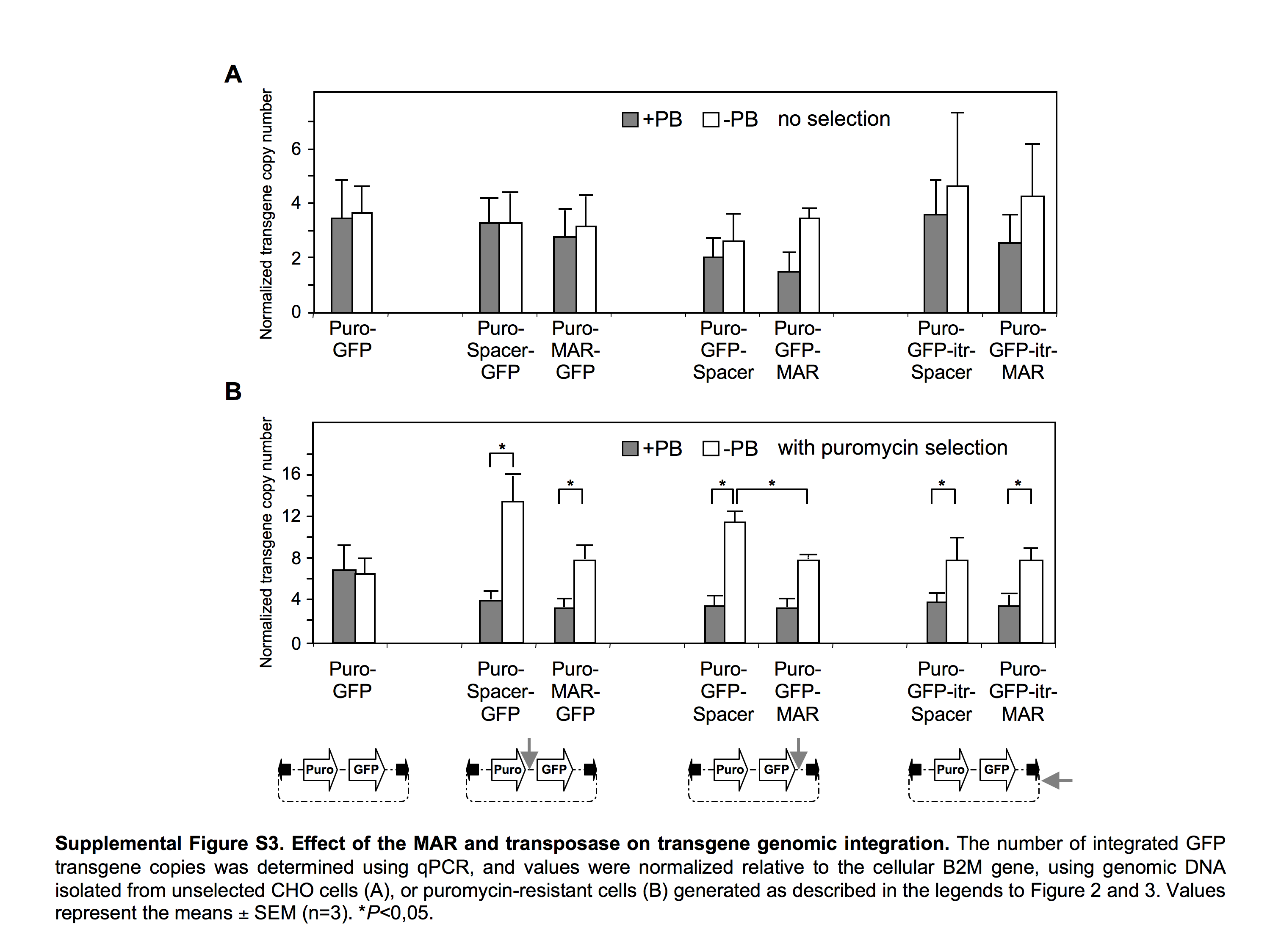

Supplement: Figure S3 — Effect of MAR and transposase upon integrated transgene copy number. The number of integrated GFP transgene copies was determined using qPCR, and values were normalized relative to the cellular B2M gene, using genomic DNA isolated from unselected CHO cells (A), or puromycin-resistant cells (B) generated as described in the legends to Figs 2 and 3. Values represent the means ± SEM (n = 3). *P<0,05. (TIF) [file pone.0062784.s003.tif]

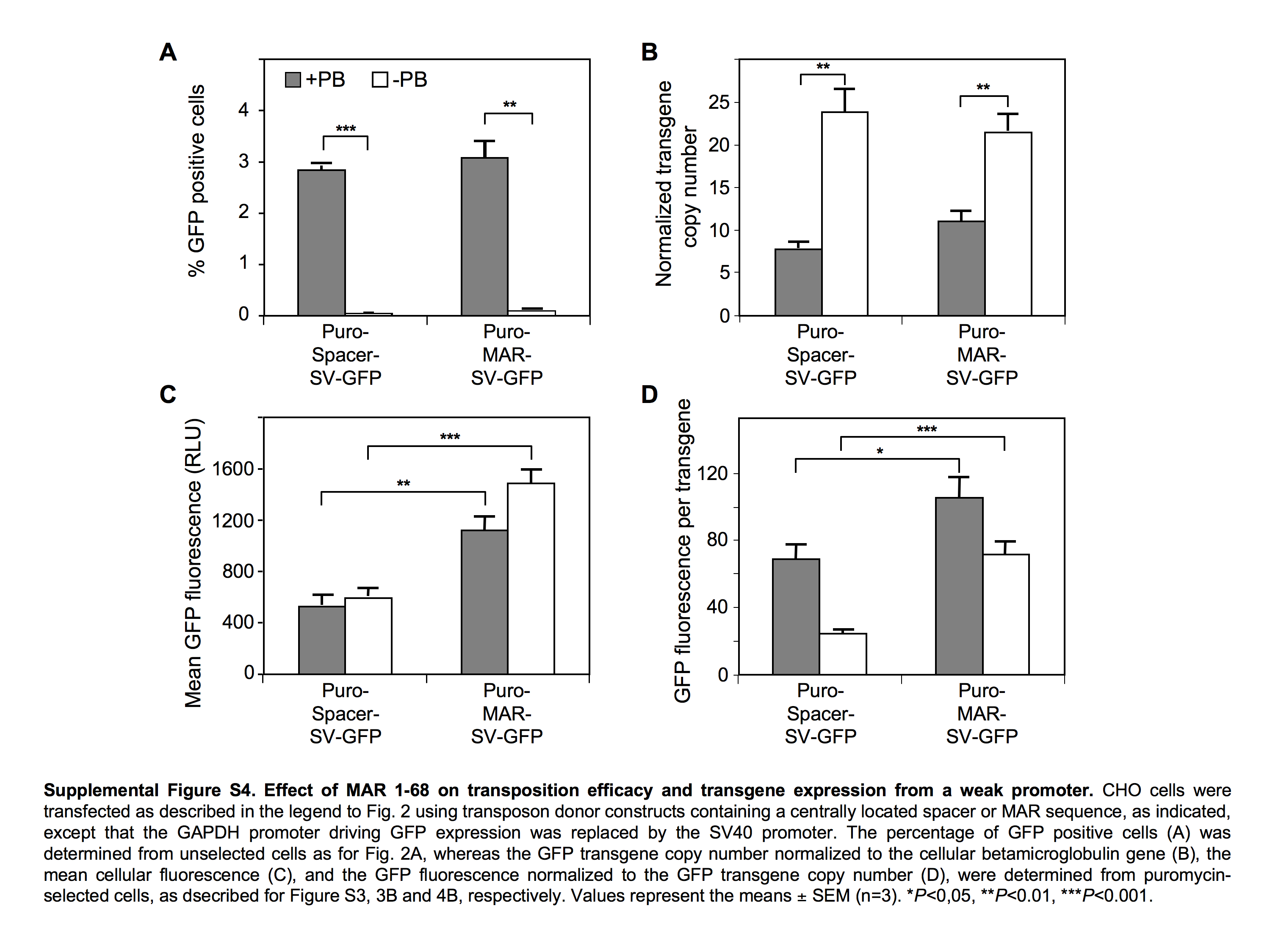

Supplement: Figure S4 — Effect of MAR 1–68 on transposition efficacy and transgene expression from a weak promoter. CHO cells were transfected as described in the legend to Figure 2 using transposon donor constructs containing a centrally located spacer or MAR sequence, as indicated, except that the GAPDH promoter driving GFP expression was replaced by the SV40 promoter. The percentage of GFP positive cells (A) was determined from unselected cells as for Figure 2A, whereas the GFP transgene copy number normalized to the cellular betamicroglobulin gene (B), the mean cellular fluorescence (C), and the GFP fluorescence normalized to the GFP transgene copy number (D), were determined from puromycin-selected cells, as described for Figure S3, 3B and 4B, respectively. Values represent the means ± SEM (n = 3). *P<0,05, **P<0.01, ***P<0.001. (TIF) [file pone.0062784.s004.tif]

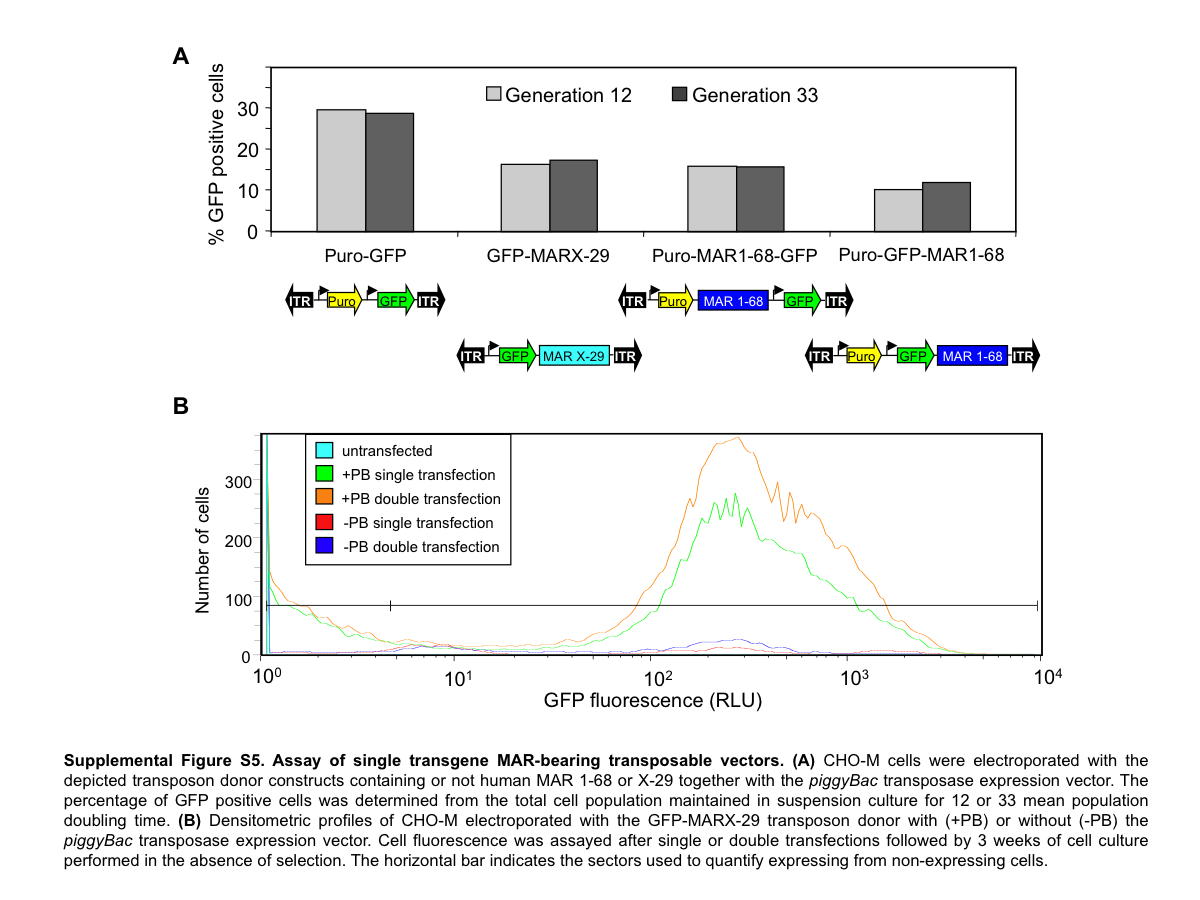

Supplement: Figure S5 — Assay of single transgene MAR-bearing transposable vectors. (A) CHO-M cells were electroporated with the depicted transposon donor constructs containing or not human MAR 1–68 or X-29 together with the piggyBac transposase expression vector. The percentage of GFP positive cells was determined from the total cell population maintained in suspension culture for 12 or 33 mean population doubling time. (B) Densitometric profiles of CHO-M electroporated with the GFP-MARX-29 transposon donor with (+PB) or without (-PB) the piggyBac transposase expression vector. Cell fluorescence was assayed after single or double transfections followed by 3 weeks of cell culture performed in the absence of selection. The horizontal bar indicates the sectors used to quantify expressing from non-expressing cells. (TIFF) [file pone.0062784.s005.tiff]

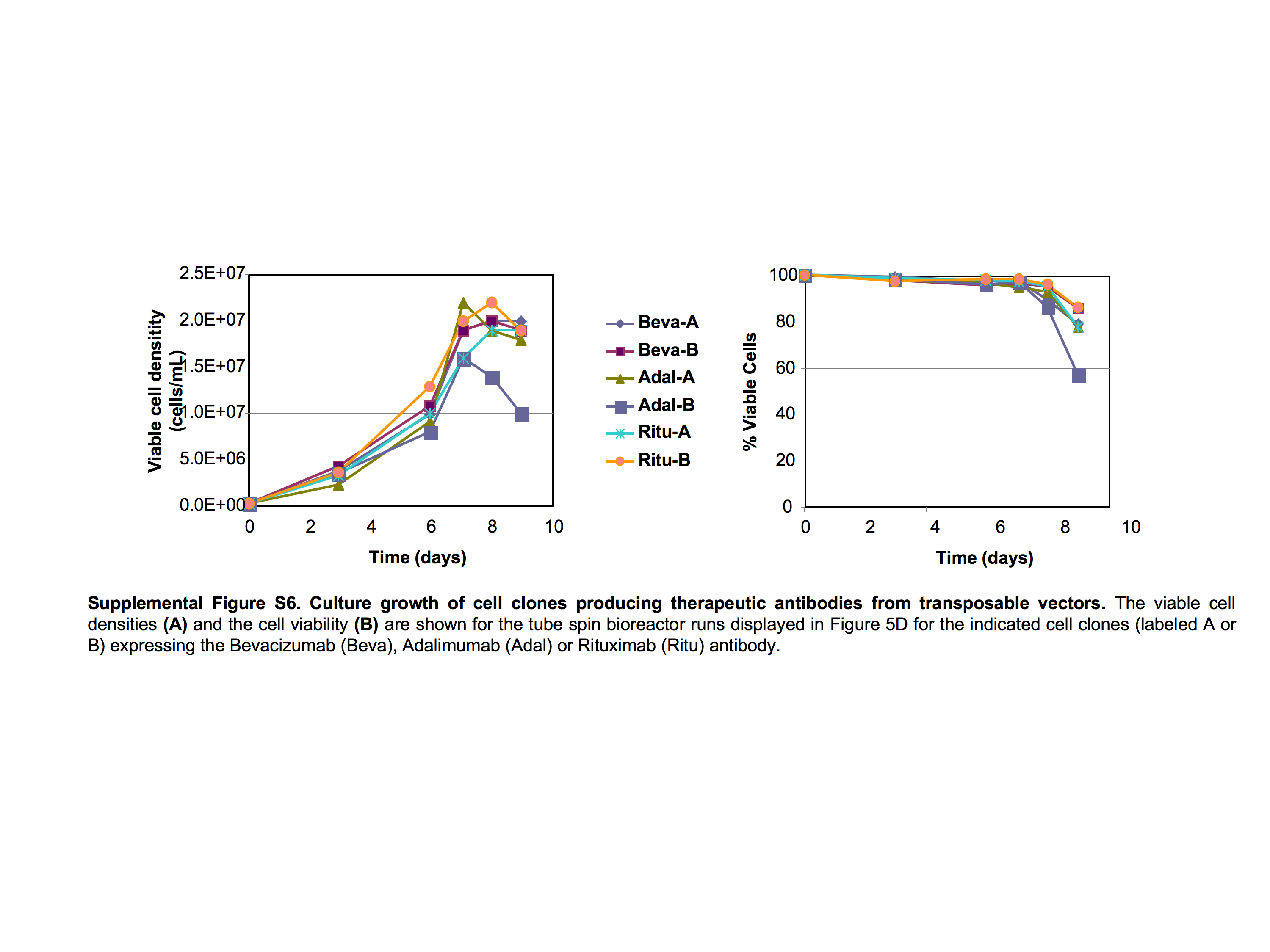

Supplement: Figure S6 — Culture growth of cell clones producing therapeutic antibodies from transposable vectors. The viable cell densities (A) and the cell viability (B) are shown for the tube spin bioreactor runs displayed in Figure 5D for the indicated cell clones (labeled A or B) expressing the Bevacizumab (Beva), Adalimumab (Adal) or Rituximab (Ritu) antibody. (TIF) [file pone.0062784.s006.tif]
